# Shape and size constraints on dust optical properties from the Dome C ice core, Antarctica

**DOI:** 10.1038/srep28162

**Published:** 2016-06-16

**Authors:** M. A. C. Potenza, S. Albani, B. Delmonte, S. Villa, T. Sanvito, B. Paroli, A. Pullia, G. Baccolo, N. Mahowald, V. Maggi

**Affiliations:** 1 Department of Physics, University of Milan, via Celoria, 16–I20133 Milan, Italy; 2 Department of Earth and Atmospheric Sciences, Cornell University, Ithaca NY, USA; 3 Department of Earth and Environmental Sciences, University Milano-Bicocca, Piazza della Scienza 1, I20126 Milan, Italy; 4 Graduate School in Polar Sciences, University of Siena, Via Laterina 8, I53100 Siena, Italy; 5 INFN, section Milano Bicocca, Piazza della Scienza, 3 - 20126 Milano Italy

## Abstract

Mineral dust aerosol (dust) is widely recognized as a fundamental component of the climate system and is closely coupled with glacial-interglacial climate oscillations of the Quaternary period. However, the direct impact of dust on the energy balance of the Earth system remains poorly quantified, mainly because of uncertainties in dust radiative properties, which vary greatly over space and time. Here we provide the first direct measurements of the aerosol optical thickness of dust particles windblown to central East Antarctica (Dome C) during the last glacial maximum (LGM) and the Holocene. By applying the Single Particle Extinction and Scattering (SPES) technique and imposing preferential orientation to particles, we derive information on shape from samples of a few thousands of particles. These results highlight that clear shape variations occurring within a few years are hidden to routine measurement techniques. With this novel measurement method the optical properties of airborne dust can be directly measured from ice core samples, and can be used as input into climate model simulations. Based on simulations with an Earth System Model we suggest an effect of particle non-sphericity on dust aerosol optical depth (AOD) of about 30% compared to spheres, and differences in the order of ~10% when considering different combinations of particles shapes.

Dust influences global climate both directly, by changing the radiative properties of the atmosphere through scattering and absorption of solar (shortwave) and terrestrial (longwave) radiation^1^, and indirectly, by impacting on cloud formation and properties^2^. While most modeling studies assume a spherical shape for dust particles^1^, detailed analyses of modern desert dust reveal significant deviations of light scattering from scattering properties of homogeneous spheres^3^,^4^,^5^ and highlight that the modeling of the light scattering by non-spherical particles is required for both accurate remote sensing and improved modeling of the climate impact of modern aerosols and dust.

For past climate conditions, ice cores offer a unique opportunity to study the optical properties of mineral dust aerosol and their role on climate evolution, as they preserve a pristine atmospheric input from the past. The EPICA-Dome C and Vostok Antarctic ice cores provide evidence of the significant increase in ice-age dust deposition flux that is inversely correlated with temperature, global sea level and atmospheric CO_2_ concentration over the last ~800 kyrs^6^,^7^. Dust could potentially play an important role in glacial-interglacial cycles^6^,^8^, but model simulations show that dust can potentially dampen or reinforce glacial–interglacial climate changes depending on its optical parameters^9^. Thus, one of the most important sources of uncertainty in paleo-climate studies is related to the poor knowledge of dust optical properties and the way they can change under different climate conditions and within a given climate period^10^,^11^.

Optical characterization of dust in ice cores is challenging^12^ because (1) the dust concentration is extremely low, especially in interglacial ice, (2) sample availability is limited if a good time resolution is desired, and (3) the particle size distribution is broad (sample polydispersity), which generally reduces size resolution. The extremely low number of particles in ice core samples limits the application of traditional light scattering approaches, except for single-particle detectors. Optical particle counters (OPC) and single particle optical sizing (SPOS, single particle obscuration sensor) are effective. The former method measures the size from the scattering cross section^13^,^14^,^15^, the latter from extinction (scattering plus absorption^16^,^17^) and it has been systematically applied in continuous flow systems for high-resolution analyses of ice cores^6^,^18^. In both cases particle size is obtained from one parameter, thus any other feature affecting light scattering remains unknown. In particular, composition, internal structure, shape and orientation of each grain can affect the measurements^19^,^20^,^21^,^22^,^23^,^24^,^25^, introducing errors in size estimates. In addition to optical methods, the Coulter Counter technique, based on electrical impedance, is widely applied in ice core measurements, mostly because of its accurate size measurement and sensitivity to low concentration levels. The Coulter Counter technique gives volume-size distributions of equivalent spherical particles without *a priori* assumptions on shape and composition. However, results from Coulter Counter and optical extinction methods often disagree, primarily because key information is missing about single particles, such as particle shape, orientation, refractive index.

In this work we applied, for the first time, the Single Particle Extinction and Scattering (SPES) method^26^,^27^,^28^ to East Antarctic ice core samples from the LGM and the Holocene. We derive information about dust particle shapes (in particular the aspect ratio), which is critical to determine the intrinsic optical properties of dust, which are needed by radiative transfer models. The SPES data, here obtained at a wavelength λ = 635 nm, consist of two terms: the extinction coefficients (*C*_*ext*_), generally plotted on the abscissa (μm^2^), and optical thickness (ρ, adimensional), plotted on the ordinate. Optical thickness is defined as ρ* = k <t> (n/n*_*0*_ −*1)*, where k = 2 π/λ (λ is the wavelength of light), *<t>* is the geometrical average thickness over the geometrical cross section in the transverse plane with respect to the incoming light, *n* and n_0_ are, respectively, the refractive indices of the particle and of the surrounding medium. The SPES method is thus able to uniquely constrain dust optical properties, which is not possible with measurements from Coulter Counter or optical extinction techniques.

## Optical Properties of Glacial and Holocene Dust From Dome C

The last climatic cycle provides a logical first target for the analysis of the paleodust optical properties from central East Antarctica, since glacial and Holocene dust size, concentration in ice, depositional flux, and sources are relatively well known^29^,^30^,^31^. On a global scale, LGM dust deposition rates were 2–4 times higher than during the Holocene^32^,^33^. In central Antarctica the observed ratios were up to ~25, mostly because of enhanced dust emission from southern South American sources and changes in dust atmospheric lifetime^34^.

In this work, we analyzed ice sections from the “*old* Dome C” ice core^35^ drilled in 1977–78 about 50 km away from the site where some years later the EPICA Dome C drilling was deployed. We analyzed samples (see Methods) from both the Holocene (H1 group ~4.2 ka BP, H2 group ~7.4 ka BP) and the LGM (G1 group ~22.3 ka BP, G2 group ~24.8 ka BP). Coulter Counter and SPES measurements were performed on two aliquots of the same sample, in order to make results fully comparable.

In order to highlight the information achievable from the SPES method, we present a subset of samples displaying very similar Coulter Counter size distribution, but very different optical properties (determined by SPES), which appreciably affect the propagation of light in the atmosphere.

**Figure 1:**
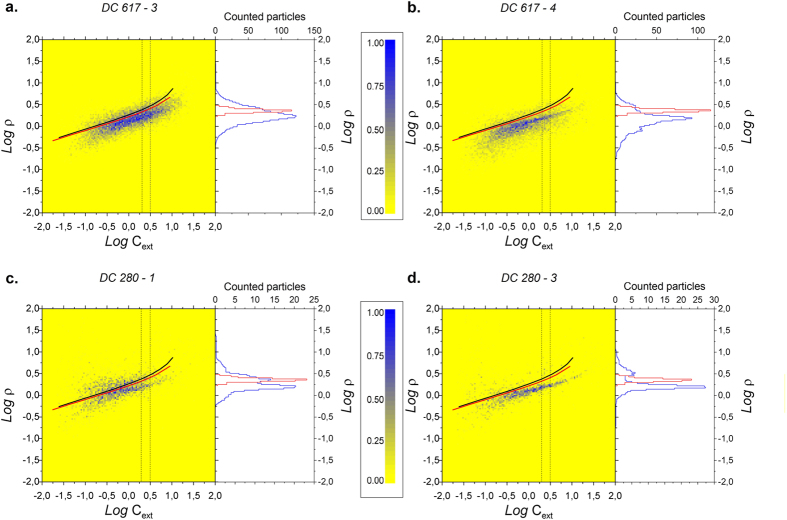
SPES optical data from the old Dome C ice core represented as the adimensional optical thickness (ρ) versus extinction coefficient (C_ext_) (μm^2^). Data are 2D histograms, the color scale indicating the number of particles (yellow = 0, blue = 1, linear scale) in each 2D bin, normalized to the maximum value of each plot. In **a** and **b** two adjacent glacial samples (LGM, ca. 22300 yrs B.P.) are considered (DC-617-3 and DC-617-4 respectively); in **c** and **d** we show two Holocene samples (ca. 7430–7440 yrs B.P.; DC-280-1 and DC-280-3). Time interval is <15 years for **a** and **b**, <10 years for **c** and **d**. Continuous lines represent the expected SPES results for spheres with refractive index n = 1.55 (black) and n = 1.50 (red), very close to the indices of the minerals found in the Dome C ice core (Gaudichet *et al*.^39^). Each plot shows the optical thickness distribution evaluated in the C_ext_ range within the vertical lines which is 2 μm^2^ < *C*_*ext*_ < 3.1 μm^2^ (blue SPES data, red simulated spheres).

In Fig. 1a,b we show SPES results obtained from two adjacent samples (DC-617-3 and DC-617-4) from the last glacial maximum (G1 group), spanning a time period of less than 15 years overall. Figure 1c,d depict the SPES plots obtained from two Holocene samples from the H2 group (DC-280-1 and DC-280-3), covering a time period of less than 10 years. Data (*C*_*ext*_ versus *ρ*) are presented as 2D histograms, the yellow-blue color scale indicating the number of particles in each 2D bin (linear scale, yellow = 0) normalized to the maximum value of each plot (blue). Continuous lines represent the SPES results expected for spheres with a refractive index n = 1.55 + 0 i (black) and n = 1.50 + 0 i (red), very close to the indices of the minerals found in the Dome C ice core^12^.

SPES data in the scatterplots are characterized by an almost horizontal dispersion, which is similar for all the samples, and is mainly driven by the size polydispersity (*i.e.* the particles size distribution) of samples^29^. On the other hand, optical thickness (*ρ*) illustrates a visible difference between samples from the same climate period (Fig. 1a vs. 1b, 1c vs 1d). Optical thickness is dependent on the (1) size, (2) effective refractive index, and (3) orientation of each particle. In order to better illustrate this difference, to the right of each SPES plot we show the distribution of the optical thickness within a given range of *C*_*ext*_ (vertical blue lines), as well as the corresponding results for the distribution of spheres (in red), for comparison. An important point to stress is that for a fixed extinction cross section (*C*_*ext*_) the distribution of the optical thickness is well described by lognormal curves whose standard deviations depend on the refractive indices of the particles as well as on their orientation imposed by shear in the liquid during the measurements (supplementary material). The range selected for C_ext_ is motivated by the appreciable broadening of data for the smallest *C*_*ext*_, (*i.e.* the smallest sizes) attributable to experimental noise. Thus, information about the shape of the particles can be extracted from the region of the plots where noise is smaller, namely for *C*_*ext*_ > 2 μm^2^ (1.1 μm in spherical equivalent diameter). Nevertheless, the whole set of data is useful for two reasons: 1) it can be directly compared to the traditional extinction measurements; 2) a very precise sizing, based upon a method which incorporates the shape effects, can be obtained by extrapolating the properties of the particles from the upper size region to the whole range. We derive information about particle shape by combining the results of numerical simulations performed for different aspect ratios, as briefly described in the supplementary material. Notice that a uniform distribution of the aspect ratio for different sizes can be reasonably assumed here, as discussed in^5^,^36^ for desert dust.

**Figure 2:**
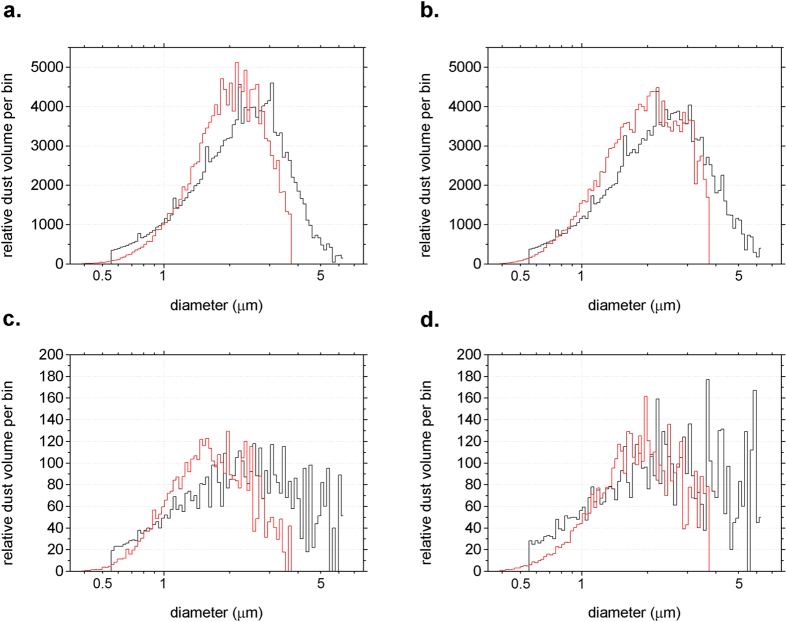
Coulter counter volume-size distributions (black) for samples shown in Fig. 1 and volume-size distributions obtained from SPES C_ext_ data (red). The SPES size range is limited between 0.5 and 3 μm (sphere-equivalent diameter).

Figure 2 shows Coulter Counter volume-size distributions of samples, performed according to the protocol adopted for the EPICA Dome C ice core^29^, and size distributions obtained from the SPES extinction data (red). These latter correspond to data which could be obtained with a traditional extinction instrument, interpreting the extinction cross sections as due to spherical particles of a given material. A specific algorithm was applied to convert extinction data into volume distributions (see Methods), to make this results as consistent as possible to earlier studies^18^. We considered the sample DC-280-3 as reference, and we used the same procedure for all the data set. Still, small differences can be observed between DC-617-3 and DC-617-4 (Fig. 2a vs. Fig. 2b), as well as between DC-280-1 and DC-280-3 (Fig. 2c vs. 2d). We interpret these discrepancies as related to different *C*_*ext*_ values for different particle shapes, for a given particle volume.

In order to interpret SPES data we rely upon a large number of numerical simulations of the radiation scattered by known, non-spherical particles with given composition, sizes, shapes, aspect ratios and orientations. Almost 10^6^ particles have been simulated and the results organized in such a way to build a benchmark of numerical results in terms of 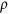 and *C*_*ext*_. This approach is well described elsewhere^37^, and some examples are reported in the supplementary material (Fig. SM4). Figure 1a,d show the typical populations obtained for oblate and prolate particles, respectively^37^. In Fig. 1b the simultaneous presence of oblate and prolate particles is evident from the superposition of two optical thickness distributions with different widths, and we can estimate a population of 75% ± 10% oblates and 25% ± 10% prolates. In Fig. 1c we have evidence of a mixture of prolate and isometric particles, as evident from the bimodal distribution of Log *ρ*, in which one of the modes is close to the sphere lines. Here a population of 60% ± 10% prolates and 40% ± 10% spheres is estimated. On the basis of the numerical simulations it is possible to extract information about the aspect ratio of dust particles. The modes of the aspect ratio distributions are 0.2 ± 0.1 (oblates, Fig. 1a) and 3.5 ± 1.3 (prolates Fig. 1d). Although the method is based upon optical measurements, strictly speaking we retrieve the geometric aspect ratio (methods).

No independent direct check is possible on these results, but independent SPES measurements have been performed on calcined standard reference materials of known composition for which precise numerical simulations for the expected shapes are possible (Fig. SM2). These results support our interpretation of ice core data.

**Figure 3:**
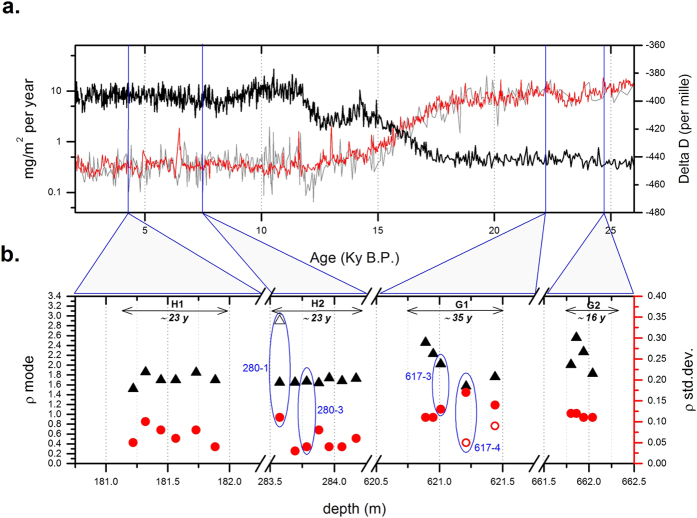
(**a**) EPICA-Dome C stable isotope profile (Jouzel *et al*.^38^; black line) and dust flux records from Lambert *et al*.^6^, and Delmonte *et al*. 2008 (Coulter Counter in grey, and Abacus in red); vertical blue lines mark the average position of the four set of samples (H1, H2, G1, G2) selected for this study from the *old* Dome C ice core. (**b**) Mode (triangles) and standard deviation (circles) of the optical thickness distributions from SPES data. The two values reported for sampleDC-617-4 (solid and open circle), are related to the presence of two populations (Fig. 1b). Ellipses evidence results obtained from samples discussed in Figs 1 and 2. Experimental errors are comparable to the size of the symbols.

In Fig. 3a we show the chronostratigraphic position of the groups of samples selected for this study from the *old* Dome C ice core with respect to the EPICA-Dome C stable isotope (paleo-temperature proxy^38^) and dust flux^6^ records. In Fig. 3b we show the mode and standard deviation of the optical thickness distributions obtained for 2 μm^2^ < *C*_*ext*_ < 3.1 μm^2^ (0.3 < Log *C*_*ext*_ < 0.5), *e.g.* corresponding to the features of the curves in the right panels of Fig. 1. Discriminating between ensembles of particles having different grain shapes is possible, in particular between samples dominated by oblate particles (DC-617-3, Fig. 1a), which reveal a large spread and a significant standard deviation in ρ, and samples characterized by a mixture of both prolate and oblate particles (DC-617-4, Fig. 1b). These latter display a distribution composed by the superposition of two lognormal curves: one characterized by a reduced spread in the vertical direction, the other by a broad vertical distribution (open and solid circle, respectively). Aeolian mineral dust at Dome C consists of a mixture of different minerals having different shapes, such as clays (illite, kaolinite, chlorite, smectite), crystalline and amorphous silica, feldspars, pyroxenes-amphiboles, metallic oxides and volcanic glasses^39^. We can therefore interpret our data as resulting from a population of particles that are preferentially oblate in some cases, and both oblate and prolate in others. Interestingly, the standard deviation of ρ is systematically higher during the LGM (average st.dev. 0.12) with respect to the Holocene (average st.dev. 0.06). This preliminary observation suggests a different shape of particles in the two climatic periods, possibly related to a different mineralogical mix. An interesting note is that preferential shape is not constant in the same climatic period, but it can vary sharply within a decade, both in glacial and Holocene climate conditions. Here we do not discuss the reason for this interesting and potentially informative high frequency mineralogical variability, but only the potential climate implications of the observed variety of shapes and associated optical properties.

### Climate Implications

Given the novelty of our approach, which provides access to properties hidden to traditional methods, we will briefly examine and discuss the corresponding effects on the airborne particles. Radiative transfer models embedded in Earth System Models usually calculate the alteration of irradiance fluxes by aerosols in the atmosphere, by combining the simulated mass mixing ratios of specific constituents and their prescribed (size- and wavelength-dependent) intrinsic optical properties (e.g. mass extinction efficiency (MEE), single scattering albedo (SSA), asymmetry parameter). Those latter can be derived off-line based on information of the particles physical properties assuming a homogeneous composition^40^.

**Figure 4:**
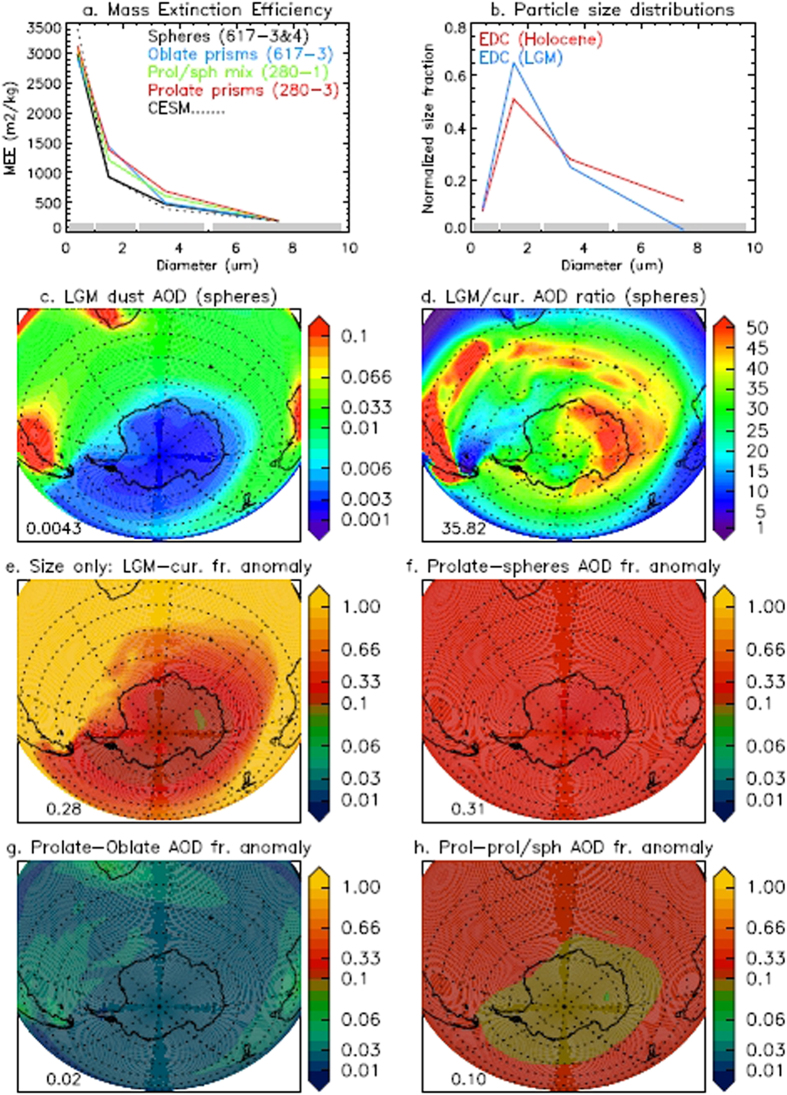
Effects of the particle size distributions and shapes on dust AOD evaluated as described in the text. (**a**) Size-resolved (on the CESM size bins, identified by the grey solid lines on the x-axis) sets of MEE (um^2^) from the SPES analyses (solid lines), and as used in the CESM [Albani *et al*.^41^] (dotted line). (**b**) Normalized particle size distributions (on the CESM size bins) from the EPICA Dome C (EDC) core for the LGM and Holocene periods (average distributions) [Delmonte *et al*.^30^]. (**c**) Atmospheric column extinction (AOD) due to dust, assuming (1) the dust mixing ratio and size distributions from a CESM simulation of the LGM climate (C4fn-lgm) [Albani *et al*.^41^], tuned to match dust deposition and size distributions at EDC, and (2) the MEE determined from SPES analyses of sample DC-617-3&4, assuming spherical particles. (**d**) LGM/current climate ratio of dust AOD, calculated assuming the dust mixing ratio and size distributions from the CESM simulations (C4fn & C4fn-lgm) [Albani *et al*.^41^], tuned to match dust deposition and size distributions at EDC, and the MEE determined from SPES analyses of sample DC-617-3&4, assuming spherical particles. (**e**) Dust AOD anomaly for changes in dust size distributions alone (comparing the LGM vs current size distributions), assuming the dust load of the tuned current climate simulation of d) and MEE of spheres (DC-617-3&4). (**f**) Anomaly (expressed as fraction) between the dust AOD based on MEE assuming prolate prisms (DC-280-3) with respect to that of spheres, assuming the same dust size distributions for current climate as in (**d**). (**g**) Same as (**f**), comparing the cases with prolate (DC-280-3) and oblate (DC-617-3) prisms. (**h**) Same as (**f**), comparing the cases with prolate (DC-280-3) prisms and a mixture (1:0.6) of prolate prisms and isometric particles (DC-280-1). Numbers in the bottom left corner of panels c-f represent the average value of the respective fields for latitudes <60 S. This figure was plotted by S. Albani specifically for this paper by means of an own-made script with IDL. IDL Version 8.2.1 (linux x86_64 m64). (**c**) 2012, Exelis Visual Information Solutions, Inc.

Different sets of values of MEE for airborne particles (Fig. 4a) were derived by numerical simulations of the particles used to interpret the data presented in Fig. 1, taking also into account aspect ratios. Different shapes have been considered (ellipsoids, cylinders, prisms with 3,4,5,6 lateral faces), but negligible differences in the optical properties were observed for a given aspect ratio; in contrast, important changes occur upon changing the aspect ratios^26^. We thus considered prisms with aspect ratios in the range 3–4 for prolate particles and 0.25–0.35 for oblate particles. No surface roughness has been included in our simulations. For comparison we also generated a reference data set for spherical particles with the same volume-equivalent size distribution, thus mimicking the Coulter Counter measurements. We consider four sets of optical properties (Fig. 4a), obtained from samples that show similar particle size distributions when measured with a Coulter Counter. This will allow us to explore and quantify the space of potential variability in the climate impacts of changing optical properties, which is hidden when not considering variability in particles shapes.

We consider the first-order impacts of different sets of optical properties derived from the SPES analyses on atmospheric dust AOD, based on dust mixing ratios simulated by the Community Earth System Model (CESM) tuned to match size and deposition at Dome C^41^ (supplementary information). In the model, CESM default values of dust optical properties have been replaced by SPES-derived optical properties, calculated for each size bin that is used in the model (4 bins). The set of MEE calculated from SPES assuming spheres is very similar (Fig. 4a) to those currently used in CESM^41^, which are based on AERONET and TOMS retrievals^42^ which assume spherical properties, giving additional confidence in our results. On the other hand, the measured samples consisting of mixtures of prolate, oblate and isometric particles (the latter approximate spheres) show substantial differences compared to the spheres, especially for particles in the 1.0–2.5 μm range. Smaller particles are more efficient scatterers, but for a given dust mass, the net effect on atmospheric extinction is modulated by the particles size distributions, which tend to be fine at the southern high latitudes (Fig. 4b). As the atmospheric dust load decreases far from the dust source areas, so does the dust AOD at 550 nm (Fig. 4c).

Next we isolate the effects on dust AOD of a realistic range of variation in dust load, size distribution and particle shape. Simulations suggest a 35-fold variability in dust AOD on the Southern Ocean and Antarctica on glacial-interglacial time scales (Fig. 4d), mostly related to variations in dust load^41^. Considering the effects of size alone (*i.e*. same total dust mixing ratios), constrained by the LGM and Holocene size distributions, model simulations suggest an increase of ~30% in dust AOD at high southern latitudes (Fig. 4e), associated with the finer size distributions observed during the LGM (Fig. 4b). The amplitude of the change in AOD due to size is the same as of considering actual particles shapes instead of assuming spheres (Fig. 4f), which shows the importance of our novel measurement. To reiterate with an example, on the basis of the numerical simulations, the average *C*_*ext*_ for the sample DC-617-3 is 3.14 μm^2^ and 4.57 μm^2^ for spheres and oblate particles respectively, whereas those same samples are very similar when only considering their extinction properties (Fig. 2c,d).

In addition, the net effect of considering the actual variability in particles shapes is also significant and can contribute changes in AOD up to ~10% (Fig. 4g,h). The effects of changing shapes is not spatially uniform: the anomalies vary as the particle size distributions shift from coarse to fine along the transport routes, highlighting again the modulation of this signal by the size distributions, which could result in sharpening or damping the gradients in dust AOD. Note that in the real world spatial and temporal variations in load, particle size distributions and shape will combine together to determine the net effects on atmospheric extinction.

Future analyses that include effects from absorption and the partitioning of back- versus forward-scattering will allow more detailed investigations of the direct impacts of dust on the atmospheric energy balance based on SPES measurements. The impact of shape variations on AOD tend to be linear with radiative forcing^8^; therefore, once regional information will be known about the shape of particles through this innovative methodology, significant changes to calculated radiative forcing will result. The important role of mineral dust aerosol in past climate changes has been widely recognized. While previous studies highlighted the role of particle shape^3^,^4^,^5^ in association to mineralogy and size in determining dust impacts on climate, we applied here for the first time the SPES method to measure dust optical properties and shape in ice cores. We anticipate that this technique will provide important constraints to climate and paleoclimate models determining the dust radiative forcing and its role on climate change.

## Methods

In this work we apply the novel single particle extinction and scattering method (SPES); it provides two independent parameters for each detected particle and is particularly efficient for very diluted and polydisperse samples such as Antarctic ice. This approach is based upon combined and simultaneous measurements of the power reduction of a laser beam in presence of the particle (i.e. extinction by very definition) and the interference between the intense transmitted beam and the much fainter forward scattered wave (i.e. scattering). In such a way it is possible to access the amplitude and phase of the scattered wave (i.e. the real and imaginary parts of the complex field amplitude^43^,^44^, see supplementary information). From SPES data each particle is thus characterized by the extinction cross section, *C*_*ext*_, and the optical thickness ρ* = k <t> (n/n*_*0*_ −*1)*, where k = 2 π/λ (λ is the wavelength of light), *<t>* is the geometrical average thickness over the geometrical cross section in the transverse plane with respect to the incoming light, *n* and n_0_ are respectively the refractive indices of the particle and of the surrounding medium. By imposing preferential orientations to the particles through shear of meltwater sample flowing into the scattering volume, we are able to evidence non-spherical particles (oblate and prolate) and to obtain a good estimate of the average aspect ratio from the measurement of a few thousands of particles (supplementary material). A benchmark of expected SPES data has been obtained from accurate numerical simulations of about 10^6^ representative particles by means of the A-DDA simulation code^45^,^46^. We have simulated the scattered fields from single particles with diameters from 0.5 to 5 μm, refractive index from n = 1.45 to 1.60, aspect ratios between 0.1 to 1 (oblate) and from 1 to 8 (prolate). For practical reasons we define the aspect ratio as the ratio between the smaller dimension and that perpendicular to it for oblate particles, and the inverse for prolates. It is thus possible to build look up tables for inferring the distribution of the aspect ratios, and therefore to achieve an accurate sizing based upon the actual shapes. Notice that, despite the optical approach, in such a way SPES partially overcomes the typical limitation of optical methods, namely to give the so called “optical diameter” and “optical aspect ratio”. Indeed, just by recovering the aspect ratio from the geometrical characteristics of the particles through proper numerical simulations, we directly provide an estimate of the geometrical aspect ratio. As a direct consequence, also the size is estimated on the basis of the true shapes, instead of the common optical size which is the diameter of a sphere having the measured cross section. These results are of great importance when extracting optical properties of the airborne particles, as discussed here.

It is impossible in general to assign one size value to a given *C*_*ext*_, as it is evident in the supplementary material Fig. SM3 for the oblate particles case. For prolates, conversely, it is easier to recover particle size thanks to a one-to-one relationship between size and *C*_*ext*_ (see Fig. 1d, DC-280-3), as explained in the supplementary material. Therefore, by following the same procedure adopted with traditional SPOS technique, we have recovered the size distribution from our extinction measurements on sample DC-280-3 (Fig. 2d, red) by assuming spherical shapes and fitting a free parameter ultimately related to the apparent refractive index, or to the optical thickness itself, to reproduce the corresponding Coulter measurements (Fig. 2d, black). The same conversion with the same parameter has been applied to the other samples. Discrepancies among Coulter and extinction in Fig. 2(a–d) can be reasonably attributed to shape effects.

The simulated dust mixing ratios, which were used as a basis for the estimation of the effects on atmospheric dust AOD of the newly retrieved optical properties, were performed with the Community Earth System Model and extensively validated for different climate conditions (including present-day and the LGM) against climate and dust observations^41^,^47^,^48^ (see supplementary material). For each horizontal grid cell in the model: 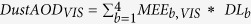 where ***b*** represents each of the four size bins in the CESM dust model (spanning 0.1–10 μm in diameter), ***MEE***_***b,VIS***_ is the size-dependent MEE in the visible wavelengths spectral band (*i.e.* 550 nm), and ***DL***_***b***_ is the column dust loading in each size class.

## Additional Information

**How to cite this article**: Potenza, M. A. C. *et al*. Shape and size constraints on dust optical properties from the Dome C ice core, Antarctica. *Sci. Rep.* **6**, 28162; doi: 10.1038/srep28162 (2016).

## Supplementary Material

Supplementary Information
