# Supplementary material for: Shape and size constraints on dust optical properties from the Dome C ice core, Antarctica
Source: Sci Rep. 2016 Jun 16;6:28162. doi: 10.1038/srep28162 (PMC4910113; doi:10.1038/srep28162)
Supplement: Supplementary Information [file srep28162-s1.pdf]

Supplementary Material of the manuscript

Shape and size constraints on dust optical properties from the Dome C ice core, Antarctica

M.A.C. Potenza[1], S. Albani[2], B.Delmonte[3], S.Villa[1], T. Sanvito[3], B. Paroli[1], A. Pullia[1], G. Baccolo[3,4], N. Mahowald[2], V. Maggi[3]

The SPES method

The SPES method[1] is based upon combined and simultaneous measurements of 1) the power reduction of a laser beam in presence of the particle (i.e. extinction by definition), and 2) the interference between the (intense) transmitted beam and the (much fainter) forward scattered wave (scattering). In Fig. SM1 a schematic of the apparatus is shown.

Let *E(r)* be the electric field of the wave radiated in the forward direction by a scatterer illuminated by a plane wave of electric field E_0. The dimensionless amplitude *S* of this field is defined as:

$$E(r) = S\frac{exp\,[ikr+ikz]}{ikr}E_0$$

where E_0 represents the amplitude of the incoming plane wave, *r* the distance from the scatterer, $k = \frac{2\pi}{\lambda}$ with λ the light wavelength. S is a complex number which real part, *R*, and imaginary part, *I*, are recovered by the SPES method[1], in contrast with traditional approaches[2]. The real part of the scattered amplitude is directly related to the extinction cross section, as described by the Optical theorem[3, 4]. This corresponds to the optical measurements performed with the traditional extinction methods[5]. The ratio between the real and the imaginary parts of the scattered field amplitude provides the optical thickness ρ of a particle with a given orientation[6], which is a measure of the amount of material in the particle that the light travels through[, 4]. Notice that, irrespectively of the geometrical cross section of a particle with a given orientation,

this parameter provides information about the degree of compactness of the particle, which in turn determines the light scattering properties.

Shapes and geometrical aspect ratio

On top of the addition information which can be obtained from the simultaneous measurements of two independent parameters through the SPES method, in this work we take advantage of giving preferred orientation to particles in the scattering region as discussed in detail elsewhere[6]. This enables a precise distinction between isometric, oblate and prolate particles, which can be further studied on the basis of specific numerical simulations.

To support this approach we present here examples of SPES results obtained with standard monophasic mineral samples of kaolinite and quartz. Accordingly to their mineralogical properties, the former is typically composed by oblate particles, the latter by prolate ones[7], as confirmed through optical microscopy and scanning electron microscopy analyses. In addition, the same samples have been characterized in the past through X-ray diffraction[8]. Therefore we can rely on standards composed by a polydisperse suspension of micron sized particles with two different preferential shapes.

These minerals have been specifically chosen in order to suspend the powders in pure water easily. No specific chemical treatment is needed to prevent the formation (nor the breaking) of aggregates, electrostatic instabilities being unimportant here. Suspensions have been prepared with concentrations which mimic the case of Antarctic dust in melted ice samples, in order to operate the SPES method in similar conditions. As a result, the number concentration of the particles is low enough to guarantee negligible aggregation. The typical aggregation time $\tau = \frac{3\eta}{4k_bT}\frac{1}{c}$ is of many days (η is the water kinematic viscosity, k_b the Boltzmann's constant, T the absolute temperature, c the number concentration of particles).

Samples are flown through a cell 0.2 mm thick in the light beam direction, but much wider in the perpendicular direction [6]. The Reynolds number is low enough (10^2) to guarantee a laminar flow, and the high (10^4) orientational Peclet number (the ratio between the velocity gradient and the rotational diffusion coefficient) shows that rotational diffusive motions are largely negligible during the time spent by a particle within the light beam (approximately 1 ms). The velocity gradient imposed by the parabolic Poiseuille flow (of the order of 10^4 s^{-1}) orientates particles along the flow over a length much smaller than the cell[9]. A strong difference thus arises among prolate and oblate particles, as depicted schematically in Fig. SM1. Prolates are oriented with the major axis transverse to the optical axis, *z*, so that light will mainly pass through the smallest thickness of the particle. By contrast, oblate particles will exhibit both major and

minor axes along *z*, and therefore the average optical thickness will be appreciably spread with respect to the prolate case.

In Fig. SM2 the SPES results on MilliQ water suspensions of kaolinite and quartz powders are reported in the ρ-C_{ext} plane adopted in this work. The histograms contain the number of particles detected in each 2D bin. The red lines represent the expected results for spheres with refractive index n = 1.5, which is representative of the index of both samples. The comparison of the two plots exhibits differences similar to those encountered in the Antarctic samples, thus proving the effectiveness of the method adopted here in distinguishing among oblate and prolate particles.

In Fig. SM3 we show the look-up table adopted for inverting data of oblate particles with n = 1.55, aspect ratios in the range from 0.15 - 1. As discussed in the Methods, for the sake of cleariness here we adopt aspect ratios smaller than 1 for indicating oblates, larger than 1 for prolates (see Fig. SM1). The color code represents the volume equivalent size r_{eq}. It is evident how different the size for a given C_{ext} can be, especially for $r_{eq} < 1$ µm. This is due to different orientations of oblate particles, and ultimately explains the discrepancies between the Coulter Counter and the extinction measurements.

We stress here that the approach we have adopted for obtaining the aspect ratios, which compares the numerical simulations of known particles to the experimental data, we can directly recover information about the geometrical aspect ratio.

Simulations of oriented, non-spherical particles

We present the results of numerical simulations obtained with particles oriented as in our measurements. In Fig. SM4.a), c) we present the results obtained for oblate particles with an aspect ratio in the range 0.15 – 0.35 and 0.35 – 0.65 respectivley and relative refractive index *n* = 1.55. Ellipsoids, cylinders, hexagonal prisms are considered. Continuous lines describe the amplitudes expected for spheres having the same refractive index as the particles.
In a similar way, Fig. SM4.b), d), show the results for prolate particles with aspect ratio in the range 3.5 – 7.5 and 1.5 – 3.5 respectively, and relative refractive index *n* = 1.55. Ellipsoids (d), cylinders (e), hexagonal prisms (f), spheres (continuous line) are considered.

Fig. SM4 e) and f) show the results obtained by mixing populations of oblates and prolates (e; 0.25 – 0.65 and 2.5 – 4.5) and isometric and provate (f; 0.75 – 1.5 and 3.5 – 7.5). The same relative abundances have

been considered. These examples support the evidence of the results obtained in Fig. 1 b) and c), where mixed populations (with different abundances) have been observed.

Simulations with the Community Earth System Model

The CESM is a fully-coupled Earth System Model, which simulates the transfer energy, mass and momentum among different components (the atmosphere, the oceans, the land surface, the cryosphere and the biosphere), using a combination of deterministic equations and physical parameterizations. The CESM participated to the Coupled Modelling Inter-comparison Project (CMIP) / Paleoclimate Modelling Inter-comparison Project (PMIP), which aims at understanding the climate sensitivity to varying forcings, and was a primary research tool for the Intergovernmental Panel for Climate Change (IPCC) Assessments Reports 4 and 5 [10] .

The dust model within the CESM [11] simulates dust emissions, transport and deposition of mineral dust. Dust emissions follow [12], and primarily depend upon winds, soil moisture and vegetation cover, as well as a geomorphic soil erodibiliy source function, which accounts for preferential sources [13]. Dust is transported as a tracer in the atmosphere, and is removed by wet and dry deposition processes. Dust impacts climate via direct radiative effects (absorption and scattering of solar and terrestrial radiation).

The simulations used as a basis for this study (C4fn for current climate, and C4fn-lgm for the LGM) use the CESM version 1.0.5, in a setup that includes the Community Atmosphere Model version 4 and the Bulk Aerosol Model, and are fully described elsewhere [14]. The Bulk Aerosol Model simulates the dust partitioning in four size bins spanning 0.1 to 10 µm in diameter, and accounts for size-dependent wet and dry scavenging processes. The simulation uses state-of-the-art parameterization of the CESM dust model, extensively optimized for current climate conditions against a wide set of observations [14].

The simulation of the Last Glacial Maximum climate uses as initial conditions an equilibrium climate simulation that followed the PMIP3 prescriptions in terms of greenhouse gases concentrations, orbital forcing, land and ice sheets, and pre-industrial vegetation [15]. In our simulation those boundary conditions were also maintained, but we accounted for changes in vegetation cover in the LGM, which is relevant for dust emissions: vegetation cover in equilibrium with the LGM climate was simulated offline with the BIOME4 model [16]. Dust emissions were then restricted to grid cells characterized by sparse or no vegetation [11].

In addition, dust emissions were spatially optimized for both climate simulations through the soil erodibility factor, in order to best fit with paleodust deposition data, i.e. a set of scale factors was applied to soil erodibility for macro-regions (broadly corresponding to the continents) in order to gain a better match to observational constraints of both mass accumulation rates and provenance fingerprinting from paledodust records [14, 18].

As a basis for this work we use the simulated size-resolved dust atmospheric mixing ratios (atmospheric concentration) from the C4fn and C4fn-lgm simulations in Albani et al. (2014) [14] . Those three-dimensional fields were adjusted for this study by a size-resolved scale factor, calculated so that model deposition matches exactly the observed average deposition and size distribution at EPICA Dome C in each climate state [17] , i.e. the mixing ratios in size bin i=1,4 at each grid cell were multiplied by the ratio (calculated for

each size bin) between the C4fn (and C4fn-lgm) simulated value of dust deposition at EDC and the observed data [17]. This correction factor maintains the coherent spatial structures of the dust mixing ratios and deposition fields as simulated by the CESM, while optimizing the fit to the observations for the specific purposes of this study, in order to explore the space of potential variability of the observationally-based dust properties. The atmospheric column extinction (dust AOD) is calculated off-line by combining the dust column load (the total mass of dust integrated above each horizontal grid cell in the model) and different values of the Mass Extinction Efficiency derived from the SPES measurements, as explained in the Methods section of the main text.

REFERENCES

1 Potenza, M. A. C., Sanvito, T. & Pullia, A. Accurate Sizing of Ceria Oxide Nanoparticles in Slurries by the Analysis of the Optical Forward Scattered Field. *J. Nanopart. Res.* **17**, 110 (2015).

2 Potenza, M. A. C. & Milani, P. Free nanoparticle characterization by optical scattering field analysis: opportunities and perspectives. *J. Nanopart. Res.* **16**, 2680 (2014).

3 Van de Hulst, H.C. Light Scattering by Small Particles. (Dover Publications, New York, 1957).

4 Bohren CF, Huffman DR (1998) Absorption and Scattering of Light by Small Particles. (Wiley & Sons, Berlin, 1983).

5 Ruth, U. Concentration and Size Distribution of Microparticles in the NGRIP Ice Core (Central Greenland) during the Last Glacial Period. PhD Thesis, Bremen University (2002).

6 Potenza, M.A.C. *et al.* Measuring shape and size of micrometric particles from the analysis of the forward scattered field. *Powder Technol*., submitted.

7. Meunier, A. (2005). *Clays*. (Springer Science & Business Media).

8 Dapiaggi, M., Sala, M., Artioli, G. & Fransen, M.J. Evaluation of the phase detection limit on filter-deposited dust particles from Antarctic ice cores. *Kristallogr. Suppl.* **26**, 73-78 (2007).

9 Gauthier, F., Goldsmith, H.L., Mason, S.G. The kinetics of flowing dispersions. *Kolloid-Z u. Z. Polymere* **248**, 1000-1015 (1971).

10 Braconnot, P. *et al.* Evaluation of climate models using palaeoclimatic data. *Nat. Clim. Chang*. **2**, 417–424, doi:10.1038/nclimate1456 (2012).

11 Mahowald, N. M. *et al.* Change in atmospheric mineral aerosols in response to climate: Last glacial period, preindustrial, modern, and doubled carbon dioxide climates. *J. Geophys. Res.* **111**, D10202 (2006).

12 Zender, C. S., H. Bian, and D. Newman. Mineral dust entrainment and deposition (DEAD) model: Description and 1990s dust climatology. *J. Geophys. Res.*, **108**, 4416, doi:10.1029/2002JD002775 (2003).

13 Ginoux, P. *et al.* Sources and distributions of dust aerosols simulated with the GOCART model. *J. Geophys. Res.* **106**, 20,255–20,273 (2001).

14 Albani, S. *et al.* Improved dust representation in the Community Atmosphere Model. *J. Adv. Model. Earth Syst.* **6**, 541-570 (2014).

15 Brady, E. C. *et al.* Sensitivity to glacial forcing in the CCSM4. *J. Clim*., **26**, 1901–1925, doi:10.1175/JCLI-D-11–00416.1 (2013).

16 Kaplan, J. O. *et al*. Climate change and Arctic ecosystems: 2. Modeling, paleodata-model comparisons, and future projections. *J. Geophys. Res.* **108**, 8171, doi:10.1029/2002JD002559 (2003).

17 Delmonte, B. *et al.* Dust size evidence for opposite regional atmospheric circulation changes over east Antarctica during the last climatic transition. *Clim. Dynam.* **23**, 427–438 (2004).

18 Albani, S., *et al.* Twelve thousand years of dust: the Holocene global dust cycle constrained by natural archives, *Clim. Past*, **11**, 869-903 (2015).

FIGURE CAPTIONS

Figure SM1: Schematic of the SPES apparatus. Water from melted ice cores is sent though a thin flow cell at a constant speed. Single particles passing through the tightly focused laser beam generate scattered waves which add to the transmitted laser beam at the sensor. Particles with different shapes are oriented in the cell thanks to the shear forces. Major and minor axes are indicated as a and b following the notation adopted in this work.

Figure SM2: Histograms show the SPES results obtained with kaolinite (a) and quartz (b) samples in the conditions described above. Red lines show the expected results for spheres. The difference between the oblate (kaolinite) and prolate (quartz) shapes is evident.

Figure SM3: The look up table adopted for inverting data for oblate particles (n = 1.55).

Figure SM4: Results obtained from numerical simulations of ellipsoids, cylinders, hexagonal prisms with relative refractive index n = 1.55; oblate shapes (a.r. in the range 0.15 – 0.35 (a) and 0.35 – 0.65 (c)) and prolate shapes (a.r. in the range 3.5 – 7.5 (b) and 1.5 – 3.5 (d)). Continuous lines describe the amplitudes expected for spheres having the same refractive index as the particles.
e): results for mixed populations of oblates and prolates (a.r. 0.25 – 0.65 and 2.5 – 4.5). f): isometric and prolate (a.r. 0.75 – 1.5 and 3.5 – 7.5). In both cases we considered a 50% abundance of each.

FIGURE SM1

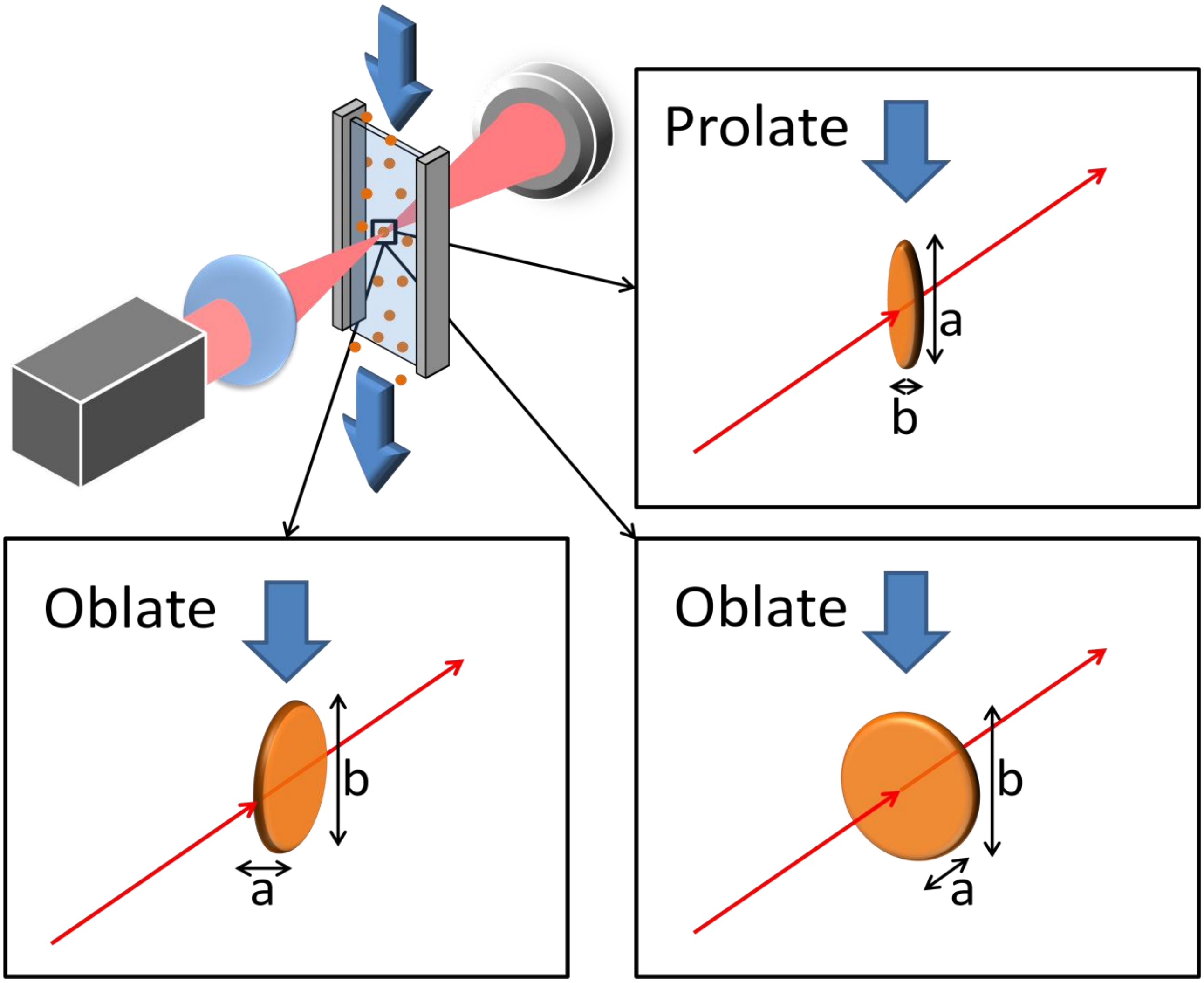

FIGURE SM2

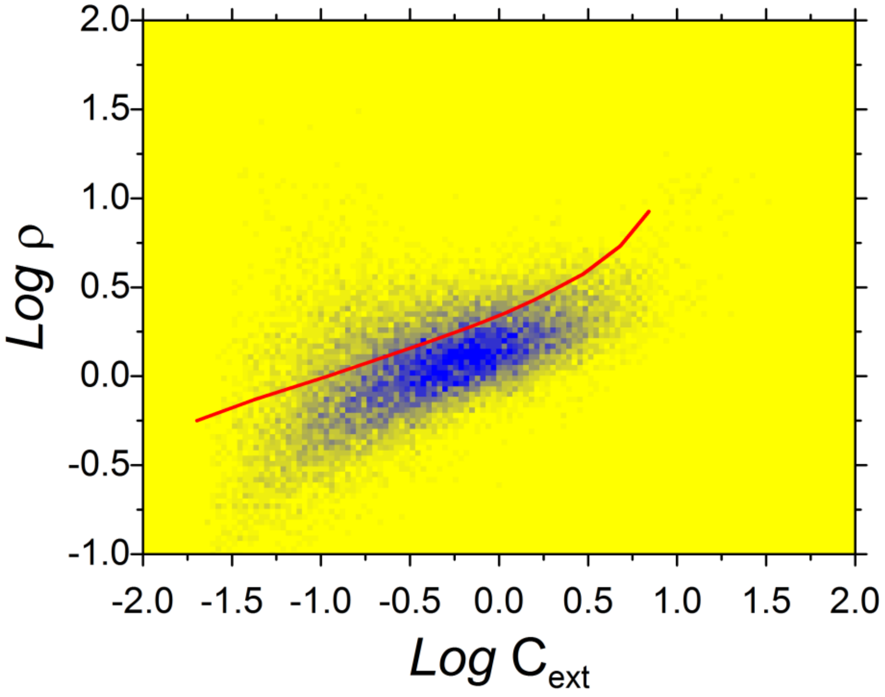
2.0
1.5
1.0
0.5
0.0
-0.5
-1.0
Log ρ
-2.0
-1.5
-1.0
-0.5
0.0
0.5
1.0
1.5
2.0
Log C$_{ext}$

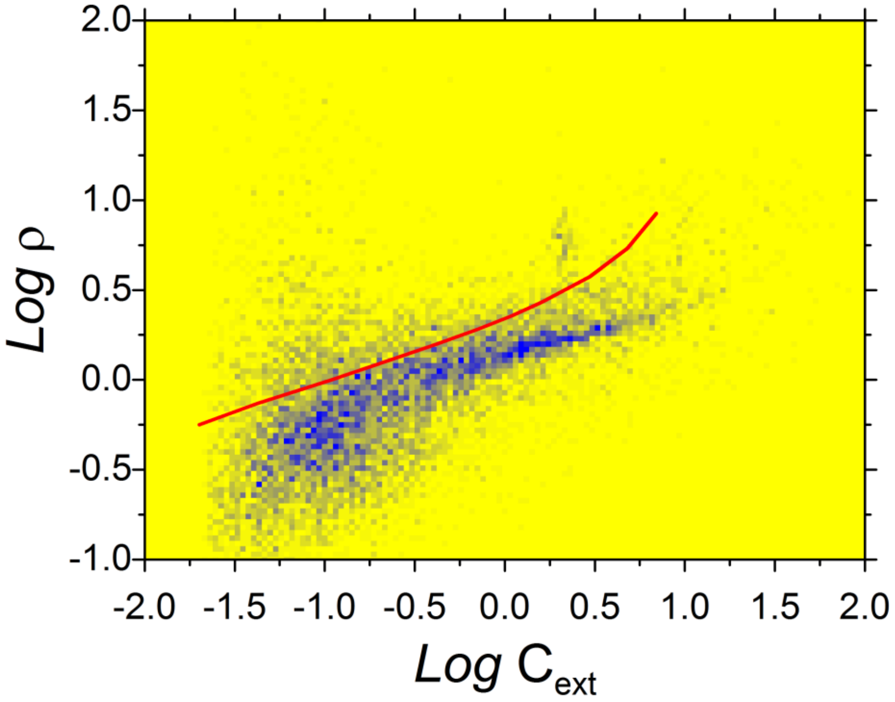
2.0
1.5
1.0
0.5
0.0
-0.5
-1.0
Log ρ
-2.0
-1.5
-1.0
-0.5
0.0
0.5
1.0
1.5
2.0
Log C$_{ext}$

FIGURE SM3

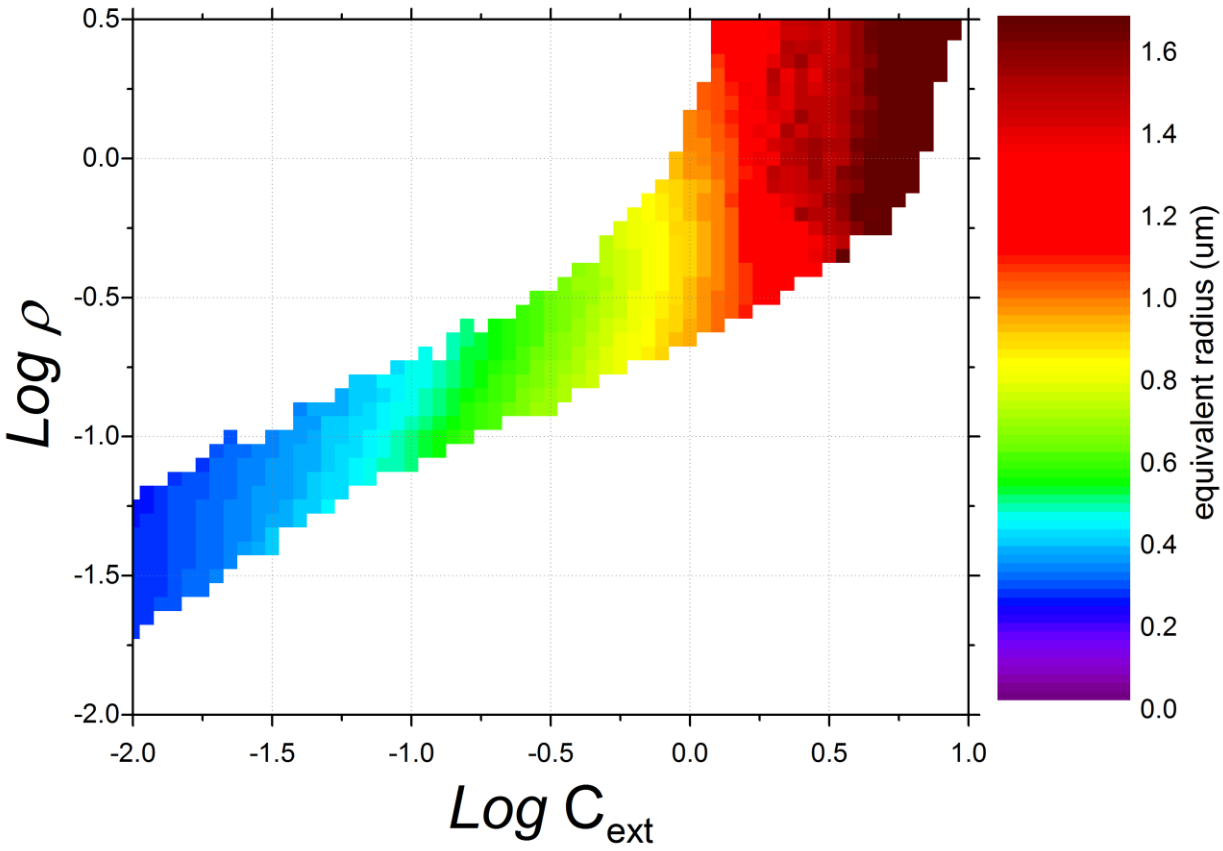

Log ρ
Log C_{ext}
equivalent radius (um)
0.5
0.0
-0.5
-1.0
-1.5
-2.0
-2.0
-1.5
-1.0
-0.5
0.0
0.5
1.0
1.6
1.4
1.2
1.0
0.8
0.6
0.4
0.2
0.0

FIGURE SM4

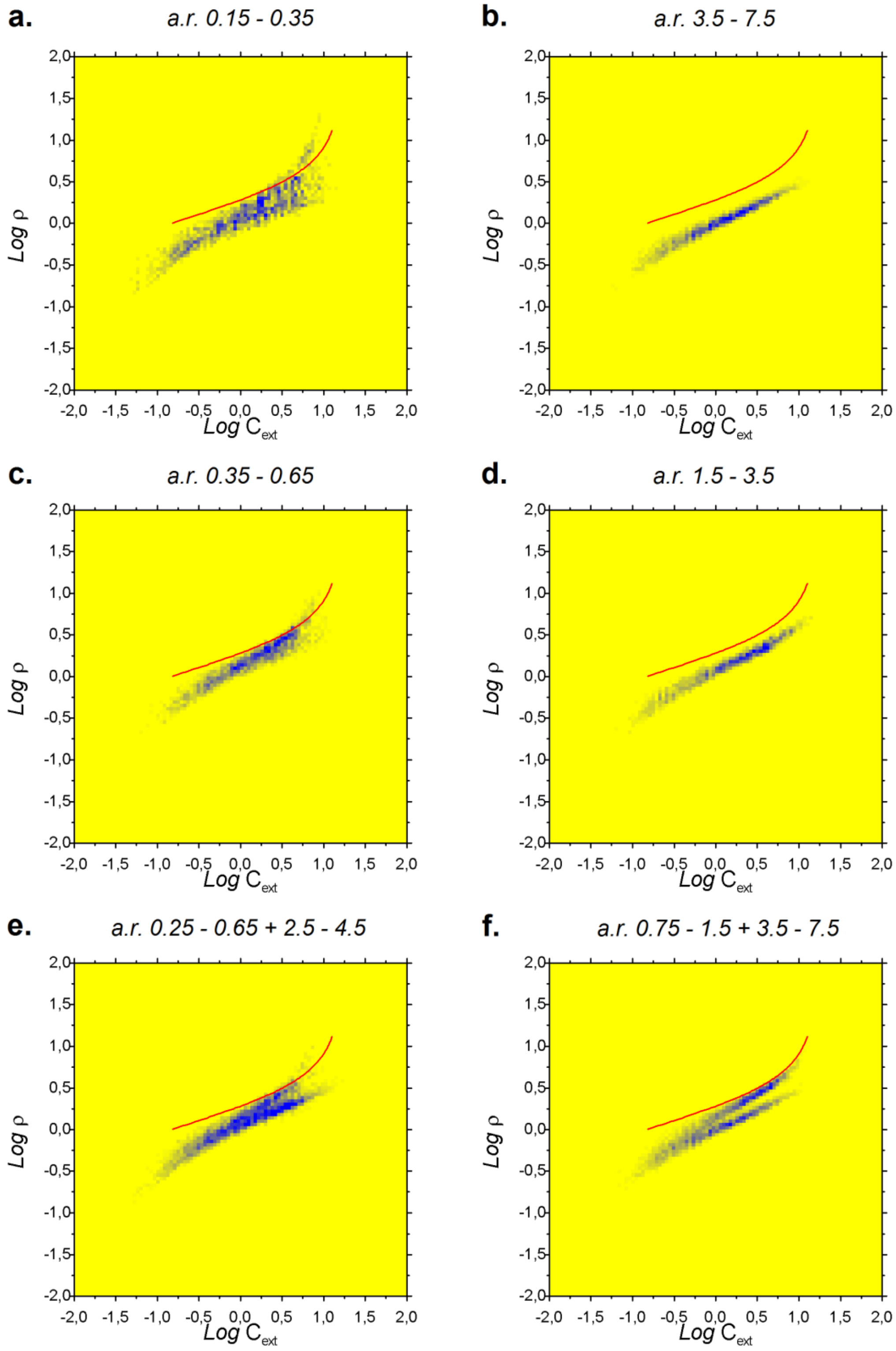
a.
a.r. 0.15 - 0.35
b.
a.r. 3.5 - 7.5
c.
a.r. 0.35 - 0.65
d.
a.r. 1.5 - 3.5
e.
a.r. 0.25 - 0.65 + 2.5 - 4.5
f.
a.r. 0.75 - 1.5 + 3.5 - 7.5
Log ρ
Log C_ext
2,0
1,5
1,0
0,5
0,0
-0,5
-1,0
-1,5
-2,0
